# Investigating seasonal association between vitamin D concentration, muscle mass and strength in postmenopausal women: a critical analysis

**DOI:** 10.1017/jns.2023.33

**Published:** 2023-07-13

**Authors:** Vrushank Shah, Hasan Zia, David F. Lo

**Affiliations:** 1 Department of Medicine, Rowan University School of Osteopathic Medicine, Stratford, NJ, USA; 2 American Preventive Screening & Education Association (APSEA), Stratford, NJ, USA; 3 Department of Biology, Rutgers, the State University of New Jersey, New Brunswick, NJ, USA; 4 Department of Medicine, Rutgers Robert Wood Johnson Medical School, New Brunswick, NJ, USA

We read with pleasure the article by Welford *et al.*^(1)^, titled ‘*Lack of significant seasonal association between serum 25(OH)D concentration, muscle mass and strength in postmenopausal women from the D-FINES longitudinal study*’ and would like to offer additional commentary on the extrapolated conclusions regarding the influence of diet, sun exposure, skin pigmentation and genetics in vitamin D serum concentrations and downstream impact on muscle mass and strength in postmenopausal women. We hope that these perspectives may provide insight into areas that may require further research and development.

First, the study did not address the effect of diet on the levels of serum vitamin D concentrations and thus on muscle mass and strength. There are several studies that show the importance of diet on serum vitamin D levels in postmenopausal women. Arikawa *et al*. state that based on the analysis of 937 postmenopausal women, the recommended intake of vitamin D (15 μg/d) was met by only 45⋅9 % of participants^(2)^. Due to the variable intake of vitamin D among participants, it is difficult to attribute changes in vitamin D concentration or the lack thereof to seasonal changes. The consumption of vitamin D-fortified foods, such as milk, butter and breakfast cereals, as well as eggs, fatty fish and mushrooms may offer good sources of vitamin D^(3)^. However, if individuals follow a vegan or vegetarian diet, then their sources of vitamin D may be limited, leading to insufficient concentrations of vitamin D. As a result, a questionnaire or survey obtaining information about the participants’ diet may be helpful to help isolate the relationship of vitamin D levels with seasonal variation and remove confounding factors.

Moreover, the amount of sun exposure along with levels of skin pigmentation should be considered as a potential variable impacting vitamin D serum concentration. There are many studies describing the conversion of sunlight (UVB solar radiation) into vitamin D3 that can be converted into the active form of vitamin D measured in blood levels. Depending on the season, participants may be more likely to perform outdoor activities and increase sun exposure, which may influence their ability to synthesise vitamin D. The level of skin pigmentation, due to melanin, is crucial for determining the amount of sunlight that can be absorbed through the epidermis. Several studies have shown that heavily pigmented skin (attributed to high levels of melanin) allows only a small amount of UVB radiation to penetrate that skin, while individuals with low levels of melanin can absorb a greater amount of UVB rays. This was proven when adult whites and adult blacks were exposed to identical amounts of UVB radiation, and the adult whites had higher blood levels of vitamin D3 by 30-fold, compared to the adult blacks, who did not show any increases in their vitamin D3 levels^(4)^. Therefore, in future studies, sun exposure levels should be quantified to determine how much vitamin D synthesis could be attributed to UVB radiation and if seasonal variations impact the level of sun exposure. Additionally, research participants with varying levels of melanin should be studied to ensure validity of results in all populations.

Finally, the study did not mention genetic variants that may influence storage and metabolism of vitamin D. Large genome-wide association studies demonstrate genetic differences leading to variability in serum vitamin D concentrations. Genetic variants related to cholesterol synthesis (*DHCR7*), conversion of vitamin D to 25(OH)D (*CYP2R1, CYP24A1*) and vitamin D transport and binding protein (*GC*) have been linked to a higher risk of vitamin D deficiency in individuals of European descent^(5)^. Moreover, genes such as CYP2R1 and SEC23A that are involved in conversion and processing of vitamin D may be susceptible to changes in season, while carriers of vitamin D lowering alleles may be unaffected by seasonal changes. The same study by Hyppönen *et al.* found that individuals with alleles of the GC locus discussed earlier may have a larger increase in vitamin D if given fortified foods and exposed to UVB rays, compared to vitamin D supplementation^(6)^. Future studies are needed to decipher genetic influences on vitamin D concentrations and seasonal changes, which may help develop more personalised treatment options for individuals with chronic insufficient vitamin D serum levels.

In the end, we applaud the authors for synthesising a multifactorial study. We look forward to reading about future studies that provide insight into the factors discussed above.
